# Malignant peripheral nerve sheath tumor in neurobifromatosis type-1: two case reports

**DOI:** 10.4076/1757-1626-2-7612

**Published:** 2009-06-09

**Authors:** Christos Kosmas, George Tsakonas, Katerina Evgenidi, Argyris Gassiamis, Lefkothea Savva, Nikolaos Mylonakis, Athanasios Karabelis

**Affiliations:** Department of Medicine, 2^nd^ Division of Medical Oncology, “Metaxa” Cancer HospitalPireasGreece

## Abstract

**Introduction:**

Malignant peripheral nerve sheath tumors are rare soft tissue sarcomas. They are considered to carry a poor prognosis with current therapeutic approaches. Successful treatment depends on a multimodal approach.

**Case presentation:**

The authors report two cases with malignant peripheral nerve sheath tumors arising from pre-existing neurofibromas in the grounds of neurofibromatis-type I. Complete surgical removal of all lesions is considered before and after induction chemotherapy. Correlation of the response to chemotherapy in the context of the immuno-histopathological features of the tumors is also discussed with reference to the existing literature.

**Conclusion:**

A need for a multidisciplinary approach with chemotherapy, surgery and radiotherapy is anticipated in the management of malignant peripheral nerve sheath tumors as described in these two reported cases. It is felt that further research on the molecular aspects of malignant peripheral nerve sheath tumors and neurofibromatis-type I will optimize treatment strategies in the future.

## Introduction

Neurofibromatosis (NF) is an autosomal dominant disorder of neural crest origin affecting all three germinal layers. It can therefore involve in any organ system. Clinically two distinct types are recognized; NF-type 1 (NF1), or von Recklinghausen disease affecting 85% of patients, and NF-type 2 (NF2), or bilateral acoustic neuromas/schwannomas affecting 10% of patients [1]. NF1 is characterized by the presence of multiple neurofibromas that may affect any organ. Discrete cutaneous and/or subcutaneous neurofibromas may develop at any age, but they occur infrequently before adolescence, varying in numbers from a few lesions to hundreds/thousands all over the body, continuing to develop throughout life [2].

Neurofibromas in NF1 may undergo malignant degeneration in 3% of patients. Schwannomas are composed entirely of Schwann cells, and malignant transformation is extremely rare; however, when it occurs, it is associated with von Recklinghausen disease in 75% of patients. NF1 is associated with an increased incidence of malignant neoplasms at any age, with a predominance of intracranial neoplasms such as, optic tract gliomas, cerebral gliomas, cranial nerve schwannomas, hamartomas, and craniofacial plexiform neurofibromas [3].

Malignant peripheral nerve sheath tumors (MPNST) constitute a heterogeneous group of malignant tumors that probably arise from cells of the peripheral nerve sheath and are categorized as soft tissue sarcomas with an associated poor prognosis and in general, limited treatment options. Factors contributing to tumor progression remain largely unknown and undefined. They represent one of the most frequent non-rhabdomyosarcomatous soft tissue tumors in pediatric age and usually occur in young adults from a previously anticipated plexiform neurofibroma in the context of NF1, with a noted change in size and pain [4]. At present there are only limited data based on anecdotal reports regarding the occurrence of MPNST in NF1 in children and adults [4,5]. Herein we report two cases of adult MPNST in NF1 and emphasize the need of a multidisciplinary approach in the treatment of these tumors.

## Case presentation

### Case Report 1

**Figure 1.**
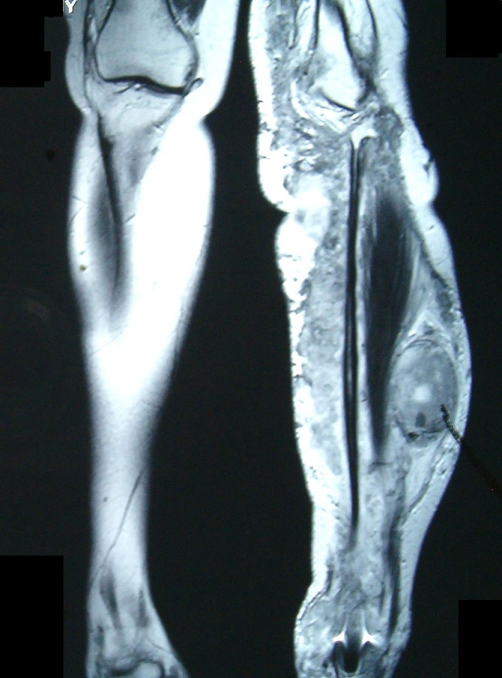
MRI image showing the site of the tumor in the left tibia (Case 1).

**Figure 2.**
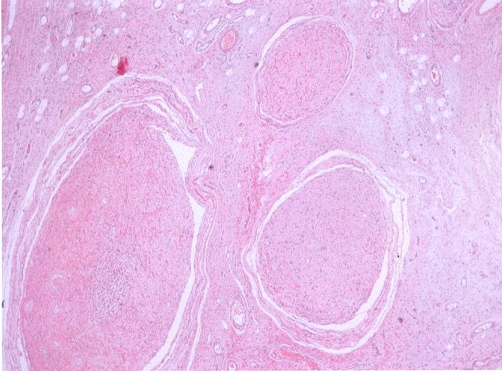
Histology demonstrates a plexiform malignant peripheral nerve sheath tumor; hematoxylin-eosin stain × 40 (Case 1).

A 23-year-old white female with a previous known history of NF1 was admitted in August 2004 after having noticed a painful, enlarging mass in her left lower leg, which rapidly increased in size within the last two months. An MRI was performed in December 2004 and revealed an extensive plexiform neurofibroma in the lower leg extending from the superior margin of the distal thigh to the ankle with areas of cystic necrosis and hemorrhage (Figure 1). However, on the MR appearances it was not fully possible to exclude malignant transformation. The biopsy revealed a high-grade spindle cell sarcoma with features consistent with a malignant peripheral nerve sheath tumor (Figure 2). Immunohistochemical analysis of the tumor specimen revealed positivity for vimentin, S-100, p53, and CD56, weak focal expression of CD117, and 20% positivity for MIB1, whereas staining for desmin, CD34, CD57, and topoisomerase IIa (TopoIIa) yielded negative results (Table 1). In January 2005, the patient presented to our hospital and a chest CT revealed a second tumor in the vertebral costal of the 11^th^ right rib extending to the adjoining bone and muscles, representing a second primary tumor. She immediately started neo-adjuvant chemotherapy with ifosfamide 2 gm/m² and doxorubicin 60 mg/m²/day × 3 days followed by cisplatin 100 mg/m² and doxorubicin 60 mg/m² every three weeks. Dose reduction in all regimens was necessary because of grade IV neutropenia. The patient completed four cycles and a new MRI revealed minimal reduction in size of both tumors, which did however, made surgical operation possible. In April 2005, she was submitted to a surgical removal of the tumor in her leg. The adjoining bone, nerve, and vascular structures remained intact. The biopsy confirmed the presence of an 18 cm MPNST with pathological features of intense mitotic activity and nuclear atypia, vascular infiltration with surgical margins being free of tumor. The patient recovered uneventfully after surgical removal of the leg lesion and was planned to be submitted to a second operation for the excision of the lesion in the chest. However, two months later she presented in the emergency room with acute respiratory failure with chest X-rays revealing rapid enlargement of the thoracic mass compressing mediastinal structures. She developed a cardiac arrest and died despite intensive resuscitation efforts.

**Table 1.** Immunohistochemical staining in resected MPNST of a 23-year-old female patient

Vimentin	Positive
S-100	Positive
CD56	Positive
CD117	Weak-focal expression
MIB-1	20%
P53	Positive
Desmin	Negative
CD34	Negative
CD57	Negative
TopoIIa	Negative

### Case Report 2

**Figure 3.**
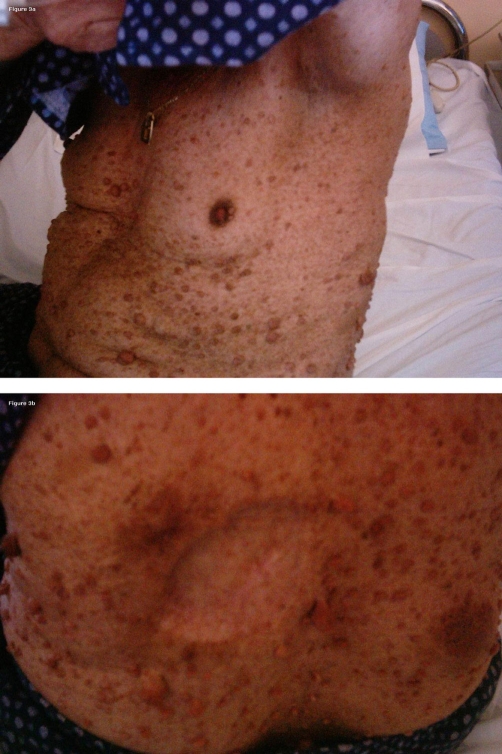
**(A)** Multiple cutaneous neurofibromas over the anterior thoracic and abdominal wall and *café-au-lait* spots characteristic of neurofibromatosis type-1 (NF1) and an axillary tumor. **(B)** Multiple cutaneous neurofibromas and *café-au-lait* spots over the dorsum and an ulcerating mass, which on biopsy revealed sarcomatous transformation (Case 2).

A 70-year-old white male with a previous history of NF1 was referred to our hospital in February 2003, where physical examination revealed the typical neurofibromas over the skin, the anterior and posterior chest and abdominal walls and *café-au-lait* spots (Figure 3A) and in addition, an ulcerated subcutaneous lesion in his upper dorsum measuring 6 × 6 cm, that had been growing slowly over the past year (Figure 3B). A thoracic CT scan and Tc-99 m-depreotide scintigraphy (Neospect) were performed revealing a second lesion in the left axillary area, apparently representing an infiltrated lymph nodal block. The FNA from the dorsal lesion demonstrated malignant sarcomatous cells. The patient underwent a surgical excision of both lesions and biopsy confirmed the presence of a spindle cell sarcoma with features consistent with a malignant peripheral nerve sheath tumor. A year later, the patient was admitted to our department for his follow-up. CT and Neospect examination indicated a relapse in the area of both surgical excisions as well as a new mass in the right lateral body of the first thoracic vertebra. He started chemotherapy with carboplatin* AUC6* on day one and etoposide 120 mg/m^2^/day on days 1-3, every three weeks. After the completion of eight courses with this regimen the patient achieved stable disease (SD). No grade 3 or 4 myelosuppression was noticed. The patient continued chemotherapy with ifosfamide 2 gr/m^2^/day over days 1-5, alternating every three weeks with cisplatin 100 mg/m^2^ and doxorubicin 60 mg/m^2^ both administered on day one and recycled every 3 weeks × 4 cycles. Grade 4 neutropenia was observed after cisplatin-doxorubicin administration, however no dose reduction was carried-out. A new radiographic evaluation performed in May 2005 showed complete eradication of the tumors in the dorsum and spine and a significant size reduction of the axillary nodal block. Thereafter, the patient received chemotherapy with carboplatin AUC2 and paclitaxel 40 mg/m^2^ administered once weekly concurrently with radiotherapy on all sites of residual disease for a total of four weeks. After the end of chemoradiation, complete remission (CR) was achieved. Eight months later, the patient developed multiple lung metastases and dyspnea. He was not subjected to further chemotherapy and died from progressive disease in the lung.

## Discussion

Malignant peripheral nerve sheath tumor (MPNST) represents a dramatic complication of NF1 and approximately 2-5% of patients with NF1 develop MPNSTs usually arising from pre-existing plexiform neurofibromas (see Figure 1). The minimum histological examination in order to establish diagnosis includes immunohistochemical stains for desmin, myogenin, vimentin, S-100, proliferative activity marker MIB-1 and potentially expression of p53, cerbB2, p27, p16 oncogenes or tumor suppressor genes. Recent studies highlight the role of TopoIIa and CD117-overexpression in certain cases as important markers for the potential administration of TopoIIa inhibitors like etoposide and doxorubicin and inhibitors of KIT-like imatinib mesylate (Gleevec) [6-8]. Radiotherapy provides local control and may delay the onset of recurrence but has little effect on long term survival. Chemotherapy has its own place in the treatment of metastatic disease. Effective drugs include ifosfamide and doxorubicin although carboplatin and etoposide have been used with promising results in metastatic MPNST refractory to first-line therapy [9]. Moreover, it may be useful in the neo-adjuvant setting in order to achieve tumor regression in patients with unresectable primaries.

In case 1, we analyzed our patient's original tumor after surgical excision with immunohistochemistry (see Table 1). The results correlated with recent studies suggesting that expression of p53 plays an important role in the evolution of MPNST from NF1 [10-12]. Furthermore, the absence of immunohistochemical reaction against CD34 is in correlation with previous findings that its expression seems to be lost during the process of MPNST formation [12]. Interestingly, the lack of expression of TopoIIa could explain the poor response to chemotherapy, in particular to doxorubicin and etoposide (TopoIIa-targeting cytotoxic drugs). More importantly, the administration of neo-adjuvant chemotherapy resulted in evasion of amputation and permitted a wide excision even though the removal of the lesion was not with completely tumor-free margins. Function of the limb was restored to normal and subsequently performance status has increased significantly, before patient's deterioration and death as a result of progression of the chest lesion.

In case 2, complete remission was achieved in our patient after a multimodal approach. A successful surgical excision of all lesions with tumor-free margins was feasible in the first place and this approach remains the cornerstone of management of MPNST. Chemotherapy with carboplatin and etoposide provided no benefit in disease control leading to SD. This is in contrast to a previous report, showing promising results with this combination as first-line therapy. A possible explanation for this lays in the fact that immunohistochemistry of our patient's biopsy demonstrated diffuse lack of expression of TopoIIa marker with only focal, positive expression in 10-20% of neoplastic cells. However, no data exist to date regarding any correlation between TopoI or TopoIIa level of expression and drug efficacy. The administration of ifosfamide plus doxorubicin appeared favorable and should be considered as the mainstay chemotherapeutic regimen of this disease as pertains to other common soft tissue sarcomas. Moreover, radiotherapy had an important role in improving local control in the areas with minimal residual disease.

In conclusion, the currently described cases emphasize that MPNSTs represent rare tumors that often occur in patients with NF1. Surgical resection represents the mainstay of treatment with consideration of neoadjuvant chemotherapy pre-operatively in order to enhance resectability [13]. Radiation and chemotherapy have a role in selected patients with MPNST and anecdotal reports with high-grade histology stress-out the potential benefits of this treatment option [14,15]. There are many recent advances in the understanding of the molecular pathogenesis of MPNST, which represent the best opportunities to develop new strategies with targeted agents and chemotherapy for the management of these patients [16-18].

## List of abbreviations

CR: Complete remission
MPNST: Malignant peripheral nerve sheath tumor
NF1: Neurofibromatosis type-1
PR: Partial remission
SD: Stable disease
topoI: topoisomerase-I
topoIIa: topoisomerase-IIa
